# Changes in the racial disparity in breast cancer mortality in the ten US cities with the largest African American populations from 1999 to 2013: The reduction in breast cancer mortality disparity in Chicago

**DOI:** 10.1007/s10552-017-0878-y

**Published:** 2017-03-08

**Authors:** Dominique Sighoko, Anne Marie Murphy, Bethliz Irizarry, Garth Rauscher, Carol Ferrans, David Ansell

**Affiliations:** 1 Metropolitan Chicago Breast Cancer Task Force, 300 S. Ashland, Suite 202, Chicago, IL 60607 USA; 2 Department of Health Systems Management, Rush University Medical Center, 1700 W. Van Buren Street, Chicago, IL 60612 USA; 3 Division of Epidemiology and Biostatistics, School of Public Health, University of Illinois at Chicago, 1603 West Taylor Street, Chicago, IL 60612 USA; 4 Department of Biobehavioral Health Science, College of Nursing, University of Illinois at Chicago, 845 S. Damen Avenue, Chicago, IL 60612 USA; 5 Department of Internal Medicine, Center for Community Health Equity, Rush University Medical Center, 600 S. Paulina, Suite 364, Chicago, IL 60612 USA

**Keywords:** Breast cancer, Mortality, Disparities, Trends, Chicago

## Abstract

**Purpose:**

Assess progress made to reduce racial disparity in breast cancer mortality in Chicago compared to nine other cities with largest African American populations and the US.

**Methods:**

The Non-Hispanic Black (NHB) and Non-Hispanic White (NHW) female breast cancer mortality rates and rate ratios (RR) (disparity) were compared between 1999 and 2005 and 2006 and 2013.

**Results:**

Between the two periods, the NHB breast cancer mortality rate in Chicago decreased by 13.9% (95% CI [−13.81, −13.92] compared to 7.7% (95% CI [−7.52, −7.83]) for NHW. A drop of 20% in the disparity was observed, from 51% (RR: 1.51, 95% CI [−7.52, −7.83]) to 41% (RR: 1.41, 95% CI [1.30, 1.52]). Whereas from 1999 to 2005 Chicago’s disparity was above that of the U.S., from 2006 to 2013, it is now slightly lower. For the remaining nine cities and the US, the mortality disparity either grew or remained the same.

**Conclusions:**

Chicago’s improvement in NHB breast cancer mortality and disparity reduction occurred in the context of city-wide comprehensive public health initiatives and shows promise as a model for other cities with high health outcome disparities.

## Introduction

Over the past twenty years, mortality related to breast cancer has fallen significantly nationwide [1]. However, disparity in Non-Hispanic black (NHB) compared to Non-Hispanic white (NHW) breast cancer mortality has been growing across the United States since the 1990s with wide geographic variability today [2–4]. In the most recently available data (2010–2014 time period), NHB women are 43.1% more likely to die from breast cancer compared to NHW women at the national level [4]. In Chicago, the growth in the disparity over the 15 years leading up to 2003 was more pronounced than the nation as a whole [5, 6]. The NHB mortality rate was 68% higher than the corresponding rate for NHW in 2003, whereas in 1991 there was no NHB/NHW mortality disparity [5].

Public concern regarding this growing disparity in Chicago led to the creation in 2007, of a task force, which became known as the Metropolitan Chicago Breast Cancer Task Force (MCBCTF). The MCBCTF called to action over 100 community/healthcare partners from 74 organizations and convened working groups for nine months [7]. Informed by Chicago-based research, including focus groups, town hall meetings and a mammography capacity, and mammography quality survey and participants’ own experiences with the Chicago breast health system, along with additional research that found a much lower disparity in New York City [8], the MCBCTF explored three guiding hypotheses as explanations for the racial inequity in female breast cancer mortality in Chicago: (1) black women receive fewer mammograms, (2) black women receive mammograms of inferior quality, and (3) black women receive lower quality treatment for breast cancer, once diagnosed [7]. In 2008, MCBCTF was formally incorporated as a not-for-profit organization with thirty-seven specific recommendations that arose from the Task Force deliberations to guide initial project strategies [9]. The MCBCTF embarked on broad-based public health, public policy, and quality improvement focused initiatives to reduce the NHB/NHW breast cancer mortality disparity in Chicago with the ultimate goal of eliminating the NHB/NHW breast cancer mortality disparity in Chicago [10, 11].

The purpose of this analysis is to compare the breast cancer mortality disparity in Chicago to corresponding trends in mortality disparities in other large urban areas with significant African American populations and to the United States as a whole, so as to assess progress made in reducing the racial disparity in breast cancer in Chicago, compared to other cities and the US.

## Methods

### Population

Chicago and 9 cities that had (1) at least 500,000 people in the total population and (2) the largest number of African Americans (the U.S. Census 2010 “The Black Population” Table 6), were included in the study, as well as US nationwide data. Cities selected based on these criteria were Baltimore, Dallas, Detroit, Houston, Los Angeles, Memphis, New York City, Philadelphia and Washington District of Columbia.

All breast cancer (BC) deaths coded C50.0–C50.9 using the International Classification for Disease 10 (ICD-10) between 1999 and 2013 were extracted from the National Center for Health Statistics databases. The extracted data were restricted to NHW and women. Person-years (p-years) were obtained from the US Census Bureau. Population by 5-year age groups for the individual 15 years of our study were available for the US, Baltimore, New York City, Philadelphia, and Washington District of Columbia. For Chicago, Dallas, Detroit, Houston, Los Angeles, and Memphis, the P-years were estimated using linear extrapolation and interpolation of the 2000 and 2010 population from the US Census Bureau by 5-year age group.

### Statistical analysis

The overall mortality rates were adjusted to the 2000 US standard population and were calculated by direct standardization. To assess the mortality and disparity trends over the 15 years of the study (1999–2013), the annual percentage changes (APC) with 95% confidence intervals (CI) were obtained using Joinpoint regression program [12]. The analyses were then stratified into two time periods: 1999–2005, which corresponds to the period before the mortality gap was reported in Chicago and 2006–2013, which includes years following the first Chicago report on the disparity. The disparity was assessed using the NHB/NHW rate ratio (RR) with 95% CI, for these two periods. The percent changes with 95% CI were calculated for the two periods. As another measure of disparity, the mortality risk difference (RD) and 95% CI were calculated across the two time periods. Excess deaths among NHB stemming from the NHB:NHW disparity were obtained by applying the age-specific NHW breast cancer mortality rate to the age-specific NHB population. These were then totaled and subtracted from the NHB observed number of deaths. The difference is the excess breast cancer mortality deaths due to the disparity. In order to assess if the trend in the racial disparity in breast cancer mortality in Chicago was specific to breast cancer, we also examined disparity trends in colon cancer mortality over the same period of time. All statistical analyses were conducted with STATA.14 (StataCorp. 2015. Stata Statistical Software: Release 14. College Station, TX: StataCorp LP.)

## Results

### Annual percent change in breast cancer mortality by race (1999−2013)

During the period 1999–2013, the NHW and NHB female breast cancer mortality rates decreased for the United States as a whole, though the annual percent change (APC) in mortality declined more slowly for NHB compared to NHW women (APC: −1.4, 95 CI [−1.5, −1.2] vs. −1.9, 95 CI [−2.0. −1.8], respectively, Table 1). There was considerable variation in APC by race across the ten cities between 1999 and 2013. Seven of the ten cities experienced a decline in NHW breast cancer mortality. Three of the ten cities experienced a significant drop in NHB breast cancer mortality (Chicago, New York City, Philadelphia), while the other seven cities experienced no statistically significant decline in NHB breast cancer mortality (Baltimore, Dallas, Detroit, Houston, Los Angeles, Memphis, and Washington DC) (Table 1).

**Table 1:** Annual percentage change (APC) in mortality rate among NHW and NHB over 15 years

	Annual percentage change (APC) from 1999–2013			
	NHW	95% CI	NHB	95% CI
US	−1.9	−2.0; −1.8	−1.4	−1.5; −1.2
Baltimore	−1.6	−3.7; 0.6	−1.2	−2.5; 0.1
Chicago	−1.5	−2.6; −0.4	−1.5	−2.3; −0.8
Dallas	−1.0	−3.2; 1.2	0.2	−1.6; 2.0
Detroit	−1.5	−2.5; −0.5	0.4	−1.0; 1.9
Houston	−1.3	−2.8; 0.1	−1.0	−2.7; 0.7
Los Angeles	−2.1	−3.3; −1.0	−0.2	−1.6; 1.3
Memphis	−1.0	−3.2; −1.2	0.17	−1.6; 2.0
New York city	−2.4	−2.9; −1.9	−1.8	−2.7; −0.9
Philadelphia	−2.1	−3.7; −0.5	−1.8	−3.1; −0.6
Washington District of Columbia	−1.8	−3.5; −0.1	−1.0	−2.3; 0.2

### Change in breast cancer mortality by race (1999–2005 vs. 2006–2013)

Next, we compared mortality rates for two specific time periods: 1999–2005 and 2006–2013. For the United States as a whole, a larger breast cancer mortality rate decline was seen for NHW than for NHB women (–13.8, (95 CI [−13.45, −13.52]) vs. −10.05%, (95% CI [−10.03, −10.07]) respectively, Table 2). After excluding Detroit, (which had unstable mortality rates for NHW women due to small NHW sample size) larger breast cancer mortality rates declines were seen for NHW than for NHB women for all but one city (Chicago). Over the period 2006–2013, the NHB breast cancer mortality rate in Chicago decreased more than the NHW breast cancer mortality rate (13.9 (95 CI [−13.81, −13.92] vs. 7.7% decline, 95% CI [−7.52, −7.83]) respectively, Table 2). Chicago’s NHB breast cancer mortality rate decrease was the largest of all the cities analyzed, followed by New York and then Philadelphia.

**Table 2:** Percent change in breast cancer mortality rates (1999–2005 vs. 2006–2013), separately by race

	Non-Hispanic White					Non-Hispanic Black				
	1999–2005	2006–2013	% Change	Lower CI	Upper CI	1999–2005	2006–2013	% Change	Lower CI	Upper CI
US	25.57	22.13	13.48	13.45	13.52	35.05	31.53	10.05	10.03	10.07
Baltimore	35.06	29.99	14.47	14.15	14.80	36.35	33.00	9.23	8.84	9.61
Chicago	27.07	24.99	7.68	7.52	7.83	40.89	35.22	13.87	13.81	13.92
Dallas	24.09	21.78	9.57	9.29	9.85	39.88	36.05	9.61	9.18	10.05
Detroit	29.50	31.76	+7.65	2.46	12.84	37.26	33.88	9.07	8.93	9.20
Houston	33.38	27.75	16.85	16.63	17.08	42.42	43.29	+2.03	1.68	2.37
Los Angeles	28.76	24.68	14.18	14.13	14.23	40.53	43.24	+6.71	6.35	7.07
Memphis	25.33	20.30	19.87	19.15	20.60	39.13	41.89	+7.04	6.47	7.61
New York city	28.85	23.90	17.16	17.11	17.21	32.64	28.44	12.85	12.72	12.98
Philadelphia	32.14	26.16	18.61	18.28	18.94	38.35	34.46	10.14	9.84	10.44
Washington District of Columbia	29.42	25.43	13.58	13.05	14.12	36.52	34.55	5.40	5.08	5.72

From 1999 to 2005, the NHB/NHW breast cancer mortality disparity in Chicago increased annually by 3.9% (95% CI [0.0; 7.9]) but that trend reversed during the time period 2006 to 2013, when the mortality disparity decreased annually by −3.1% (95% CI [−5.4; −0.8]). As a result, whereas Chicago’s mortality disparity was greater than for the US during 1999–2005, Chicago’s mortality disparity was slightly lower than for the US during 2006–2013 (Table 3).

**Table 3:** NHB/NHW disparity (rate ratio) in breast cancer mortality rates, separately by calendar period (1999–2005 vs. 2006–2013)

	1999–2005			2006–2013		
	Rate ratio	Lower CI	Upper CI	Rate ratio	Lower CI	Upper CI
US	1.37	1.36	1.39	1.42	1.41	1.44
Baltimore	1.04	0.91	1.18	1.10	0.95	1.28
Chicago	1.51	1.40	1.63	1.41	1.30	1.52
Dallas	1.66	1.45	1.89	1.66	1.45	1.89
Detroit	1.26	1.06	1.50	1.07	0.87	1.31
Houston	1.27	1.15	1.40	1.56	1.42	1.71
Los Angeles	1.41	1.28	1.55	1.75	1.61	1.91
Memphis	1.54	1.34	1.79	2.06	1.79	2.39
New York city	1.13	1.08	1.19	1.19	1.13	1.25
Philadelphia	1.19	1.09	1.30	1.32	1.21	1.44
Washington District of Columbia	1.24	1.05	1.47	1.36	1.15	1.60

### Comparison of breast to colon cancer mortality trends in Chicago

We compared trends in breast cancer mortality by race in Chicago to the corresponding trends in colorectal cancer mortality during the time period 1999–2013. Colon cancer mortality rates decreased during this period regardless of race resulting in annual average reduction in the colon cancer mortality disparity of 0.3%. From 1999 to 2005, the colon cancer mortality disparity was similar to that for breast cancer; however, for the period from 2006 to 2013, the breast cancer mortality disparity decreased while the colon cancer mortality disparity remained unchanged.

### Excess number of deaths due to breast cancer for NHB women

Compared to the other nine cities and the US as a whole, NHB women in Chicago experienced the highest drop in breast cancer mortality rate in the nation, followed by New York City (Table 2). The number of excess deaths due to breast cancer for NHB women in Chicago declined by 29%: from 537 cases in 1999–2005 to 394 in 2006–2013 (Table 4). In contrast, the NHB/NHW breast cancer mortality disparity in the US as a whole grew corresponding to a 22% increase in the excess number of deaths due to breast cancer for NHB, from 12,177 to 14,897 between these two periods (Table 4).

**Table 4:** Absolute NHB/NHW disparity in breast cancer mortality rates and excess death, separately by calendar period (1999–2005 vs. 2006–2013)

	1999–2005				2006–2013			
	Rate difference	Lower CI	Upper CI	Excess death in NHB	Rate difference	Lower CI	Upper CI	Excess death in NHB
US	9.48	7.52	11.44	12,177	9.40	7.44	11.36	14,897
Baltimore	1.29	−0.67	3.25	20	3.01	1.05	4.97	77
Chicago	13.82	11.86	15.78	537	10.23	8.27	12.19	394
Dallas	15.80	13.86	17.78	182	14.27	12.32	16.24	181
Detroit	7.76	5.81	9.73	215	2.12	0.17	4.09	54
Houston	9.04	7.09	11.01	166	15.53	13.58	17.50	322
Los Angeles	11.77	9.82	13.74	172	18.57	16.62	20.54	274
Memphis	13.80	11.86	15.78	208	21.59	19.65	23.57	379
New York city	3.78	1.82	5.74	293	4.54	2.58	6.50	571
Philadelphia	6.21	4.25	8.17	156	8.30	6.35	10.27	354
Washington District of Columbia	7.09	5.15	9.07	90	9.12	7.16	11.08	183

## Discussion

There are three major observations from this study on NHB and NHW breast cancer mortality in the ten US cities with greater than 500,000 people and largest NHB populations. First, in the United States as a whole and in most of the cities included in this study, breast cancer mortality has declined for both NHB and NHW, although the NHW breast cancer mortality reduction has been steeper than that for NHB. Nationally, this difference has resulted in a growing NHB/NHW disparity with a greater NHB to NHW disparity in the period 2006–2013 compared to the years 1999–2005. Advances in breast cancer screening and treatment are having an impact across both races in most cities, but because NHB breast cancer mortality rate reductions have lagged in many cities, and in a few cities have actually risen, the health inequity has grown, nationally.

The second observation from these data is the presence of tremendous geographic variation in NHW and NHB breast cancer mortality and disparity. The reasons for the geographic variation are unclear though they are likely driven by structural issues rather than biological variation such as access to health care, including state variation in Medicaid coverage even prior to the Affordable Care Act, variation in healthcare quality, health insurance rates, structural racism, and dysfunction of the health care safety-net. A recent study on fifty US cities looking at Black/White breast cancer mortality disparities from 2005 to 2014 notes this variability [4].

In our study, the NHW breast cancer mortality rates declined for the periods 1999–2005 and 2006–2013, in every city except Detroit, where NHW breast cancer mortality rose between the two time periods. Between these two time periods, NHB breast cancer mortality declined in three cities, (New York, Philadelphia, and Chicago) and was flat or rose in the other seven major cities. These geographic variations in disparity are driven by both racial groups’ mortality rates and the relative change between them. The NHW breast cancer mortality rates across the ten cities vary by about 10 deaths per 100,000 from the lowest NHW mortality city in the period 2006–2013 (Memphis) to the highest NHW mortality city (Baltimore). The NHB breast cancer mortality rates vary by 15 deaths per 100,000 from the lowest NHB mortality city (New York City) to the highest NHB mortality city (Houston).

The third observation is that the NHB/NHW breast cancer mortality disparity in Chicago has dropped in contrast to the national trend. In the period 1999–2005, Chicago had among the highest NHB to NHW breast cancer disparity in the nation. This disparity was the result of a very high breast cancer mortality rate for NHB women, compared with a declining rate for NHW women. From 1999 to 2005, the NHB breast cancer mortality rate in Chicago was the third highest among the ten cities. In contrast, from 2006 to 2013, the NHB/NHW breast cancer disparity decreased by almost 20%, largely because of an almost 14% drop in NHB breast cancer mortality. This decline in NHB breast cancer deaths is the largest observed in the ten cities comparing the two time periods. Chicago is also the only city of the ten cities where the NHB breast cancer mortality rate dropped faster (13.9%) than the NHW (7.7%) breast cancer mortality rate. Chicago’s mortality rate decrease for NHW (7.7%) is lower than the NHW decrease in most other cities, thus also contributing to a lowering of the overall disparity in mortality outcomes in Chicago. In contrast to breast cancer disparities, analysis of NHB to NHW colorectal cancer mortality disparity showed no improvement comparing the same time periods.

Elimination of health disparities remains a major public health goal in the United States [13]. Yet, mortality disparity for breast cancer is increasing rather than decreasing [1]. However, Chicago’s sharp reduction in the NHB breast cancer mortality rate and NHB/NHW breast cancer mortality disparity stands in contrast with the national trend.

Observational time-trend mortality analyses do not lend themselves to causal explanations. However, one reason for the observed sharp reduction in NHB breast cancer mortality rate in Chicago could be the public health attention that racial disparity in breast cancer mortality has received in Chicago. In 2007, researchers and health activists made reducing the racial gap in breast cancer mortality a public health priority in Chicago. The Metropolitan Breast Cancer Taskforce was established as a not for profit dedicated exclusively to eliminating this disparity. Chicago-based research over this time period documented a wide array of structural and quality inequities, including poorer mammography quality, missed breast cancer for minority and lower-income women leading to later stage diagnosis [14–16], absence of American College of Radiology Breast Imaging Centers of Excellence and Academic/Comprehensive Commission on Cancer Accredited Cancer programs in minority neighborhoods where breast cancer mortality rates are highest [7], and breakdowns in the breast cancer diagnostic and referral process [17, 18]. The gaps led MCBCTF and others to create specific initiatives to address differential access to high quality care. A critical initiative was the creation of a metro-wide Breast Cancer Quality Consortium to engage the institutions to share quality data on breast cancer screening and treatment. Gaps in care were identified, quality improvements interventions were implemented, and improvements were made [11, 19–21].

The issue of racial disparity in breast cancer mortality was discussed widely in news outlets and community meetings [22, 23]. The Chicago Department of Public Health made the reduction of racial disparity in breast cancer mortality a cornerstone of their Healthy Chicago plan [24]. Public policy changes through passage of breast cancer disparities reduction legislation were passed (Public Law 95-1045 and Public Law 97–0638). In addition to these efforts, MCBCTF and others implemented very specific outreach, education, and patient navigation initiatives to address identified deficits and overcome the fragmented Chicago healthcare system in particular for minority women who are more likely to enter the system through a more limited service facility rather than at a comprehensive breast center [9, 25].

Even with the notable drop in NHB breast cancer mortality and disparity in Chicago, the NHB to NHW breast cancer mortality gaps remain unacceptably large in this city and in most of the other nine cities. But public health and quality improvement approaches that focus on structural inequities in health delivery like that of the Metropolitan Chicago Breast Cancer Taskforce may be a replicable model to address breast cancer disparities in other US cities.

## Conclusions

What is undeniable is that there is still tremendous variability in NHB and NHW breast cancer mortality and disparity among the cities in the US with the largest African American populations. The variability in both mortality and disparity in the NHB and NHW populations can only be explained by social and structural determinants operating at the local level. The northeast cities of New York City and Philadelphia have had among the fastest decline in NHB and NHW mortality rates, while the southern and western cities of Dallas, Houston, Los Angeles, and Memphis have had the least progress in disparity reduction. When it comes to racial disparity in breast cancer mortality, geography appears to dictate destiny. However, the public health lesson from Chicago is that these disparities may not be inevitable and might be reduced with large city-wide and state-wide initiatives, such as those implemented in Illinois since 2006 by MCBCTF and its partners.
